# Using scRNA-seq to Identify Transcriptional Variation in the Malaria Parasite Ookinete Stage

**DOI:** 10.3389/fcimb.2021.604129

**Published:** 2021-03-01

**Authors:** Kathrin Witmer, Farah Aida Dahalan, Tom Metcalf, Arthur M. Talman, Virginia M. Howick, Mara K. N. Lawniczak

**Affiliations:** ^1^ Parasites and Microbes Programme, Wellcome Sanger Institute, Hinxton, United Kingdom; ^2^ Department of Life Sciences, Imperial College London, London, United Kingdom; ^3^ MIVEGEC, IRD, CNRS, University of Montpellier, Montpellier, France; ^4^ Institute of Biodiversity, Animal Health and Comparative Medicine, College of Medical Veterinary and Life Sciences, University of Glasgow, Glasgow, United Kingdom; ^5^ Wellcome Centre for Integrative Parasitology, College of Medical Veterinary and Life Sciences, University of Glasgow, Glasgow, United Kingdom

**Keywords:** ookinete, *Plasmodium*, scRNA-seq, transcriptomics, malaria, *Anopheles*

## Abstract

The crossing of the mosquito midgut epithelium by the malaria parasite motile ookinete form represents the most extreme population bottleneck in the parasite life cycle and is a prime target for transmission blocking strategies. However, we have little understanding of the clonal variation that exists in a population of ookinetes in the vector, partially because the parasites are difficult to access and are found in low numbers. Within a vector, variation may result as a response to specific environmental cues or may exist independent of those cues as a potential bet-hedging strategy. Here we use single-cell RNA-seq to profile transcriptional variation in *Plasmodium berghei* ookinetes across different vector species, and between and within individual midguts. We then compare our results to low-input transcriptomes from individual *Anopheles coluzzii* midguts infected with the human malaria parasite *Plasmodium falciparum*. Although the vast majority of transcriptional changes in ookinetes are driven by development, we have identified candidate genes that may be responding to environmental cues or are clonally variant within a population. Our results illustrate the value of single-cell and low-input technologies in understanding clonal variation of parasite populations.

## Introduction

Malaria is a devastating mosquito-borne disease caused by single-celled apicomplexan parasites belonging to the *Plasmodium* genus. Transmission begins with ingestion of an infectious blood meal by the mosquito. This initiates the most extreme population bottleneck in the parasite’s life cycle, where sexual stage parasites must rapidly undergo fertilization, develop into invasive ookinetes, and transit through the midgut epithelium of the mosquito before beginning sporogonic development. Only a small proportion of ookinetes will successfully cross the midgut (Smith et al., 2014). Although the ookinete stage is a critical bottleneck in the life cycle, relatively little is known about the molecular and population-level processes that drive successful midgut invasion of ookinetes. We also lack information on how this life stage copes with the variable environment it encounters including for example potentially highly diverged mosquito species and also highly variable microbiota between individual mosquitoes.

Variation between individuals provides necessary material for natural selection when environmental conditions change. Perhaps the most extreme environmental changes the malaria parasite experiences in its life cycle are the transitions between mammalian and mosquito vector hosts. Additionally, the parasite must be able to cope with unpredictable variation within and between hosts in order to survive. *Plasmodium falciparum* blood stage parasite populations can deal with this host environmental variation through rapid response to cues or alternatively through phenotypic diversification or bet-hedging independently of these cues (Llorà-Batlle et al., 2019). There is evidence that malaria parasites use bet-hedging strategies in the blood stage forms to optimize survival in face of variation in the mammalian host immune response and potentially during conversion to the sexual transmissible stage parasites through epigenetic regulation that results in transcriptional variation within a clonal population (Rovira-Graells et al., 2012; Brancucci et al., 2014; Voss et al., 2014; Waters, 2016; Fraschka et al., 2018). Other work has shown that the vector plays a role in modulating virulence repertoires *via* a transcriptional reset after transmission through the mosquito (Spence et al., 2013). The examination of whether parasites employ bet-hedging strategies in the mosquito host has been less well studied.

Single-cell technologies provide the possibility to understand clonally variant transcriptional patterns in unicellular parasites by deconvoluting the contribution of each individual and allowing for precise ordering of parasites in developmental time (Poran et al., 2017; Brancucci et al., 2018; Nötzel et al., 2018; Reid et al., 2018; Howick et al., 2019). In *Plasmodium*, a large proportion of the transcriptome is differentially expressed between parasite stages through tight regulation of transcription *via* a small number of ApiAP2 transcription factors (Bozdech et al., 2003; Modrzynska et al., 2017). These large changes in expression make it difficult to understand what proportion of variation observed is a result of developmental heterogeneity in a sample versus variable patterns of expression at a single point along this developmental trajectory when using bulk transcriptomic approaches (Llorà-Batlle et al., 2019). In this study, we built a framework to minimize the effects of development in single-cell RNA-seq data by identifying the most mature populations of parasites and statistically controlling for developmental timing in our analyses.

Using this framework, we investigate variable patterns of gene expression in *Plasmodium berghei* ookinetes across two vector species as well as within and between individual mosquito midguts (**Table 1**). We identify genes that are differentially expressed across these environments as well as a subset of genes that are highly variably expressed independent of developmental time and the vector host. We then revisit bulk RNA-seq data from individual vector midguts from mated or virgin *Anopheles coluzzii* infected with *P. falciparum* (Dahalan et al., 2019) and identify variable genes that may be responding to vector host physiological factors. We find that ookinetes display subtle differences in gene expression not only based on the vector species, mating status or antibiotic treatment, but also within one mosquito individual. Our findings suggest that a fine balance between adaptive gene expression and intrinsic variant gene expression act together to ensure successful gut colonization.

**Table 1:** Overview of data sets and differential gene expression (DE) analysis performed in this study.

Data Set	*Plasmodium* species	*Anopheles* species	Sample collection	# midguts	Method	Cells/samples passing QC	DE comparison
**Pb vector species**	*P. berghei*	*An. coluzzii* *An. stephesi*	Pooled guts	5 per pool	Single-cell	253	Mosquito species
**Pb single-gut**	*P. berghei*	*An. stephensi*	Single guts	4	Single-cell	58; 67; 88; 91	Individual guts Within a gut
**Pf bulk**	*P. falciparum*	*An. coluzzii*	Single guts	24	Bulk	22	Mating status Antibiotics Individual guts

We analyzed three different data sets. First, data from the Malaria Cell Atlas (Howick et al., 2019) consisting of single cell P. berghei ookinetes collected from pooled An.stephensi midguts was compared to a data set collected simultaneously but from An. coluzzii midguts that we called Pb vector species data set. Second, we analyzed single cell P. bergheiookinetes that were collected from four individual An. coluzzii midguts and named this data set Pb single gut data set. Third, we used P. falciparum bulk RNA-seq data from a previously published data set (Dahalan et al., 2019) that investigated P. falciparum infection in An. coluzzii in mated compared to virgin females. We named this data set Pf bulk data set.

## Materials and Methods

### Retrieval and scRNA-seq of *P. berghei* Ookinetes

*P. berghei* parasites were propagated in female 6- to 8-week-old Theiler’s Original outbred mice supplied by Envigo UK. The clonal *P. berghei* RMgm-928 strain (Burda et al., 2015), which expresses mCherry under the control of the hsp70 promoter throughout the life cycle, was used for all *P. berghei* transcriptomic data. A Hap2 knock-out *P. berghei* strain in the RMgm-928::mCherry background, which does not produce male gametocytes, was used as a control for FACS gating (Howick et al., 2019). Mosquito infections were performed in two- to five-day-old *Anopheles stephensi* or *An. coluzzii* through direct feed on mice.

*P. berghei* parasites were isolated from the mosquito blood bolus at 18 or 24 h post infectious feed as previously described (Howick et al., 2019). Briefly, each midgut was dissected and a lateral incision was made along the gut epithelium to release the contents of the bolus into a small drop of PBS and the midgut tissue was further rinsed with a syringe. The released contents were transferred to an Eppendorf tube stored on ice. Each sample was diluted in an additional 400 µl of PBS, filtered with a 20 µm filter, and stained with SYBR green (1:5,000) prior to FACS. For the *Pb vector species data set*, the contents of five boli were pooled prior to sorting at 18 h (*An. stephensi*) and 24 h (*An. stephensi* and *An. coluzzii*). The 18 h time point was included in our analysis to inform developmental trajectory inference. For the *Pb single-gut data set*, parasites were collected from each gut at 24 h post-infection and maintained and sorted as separate samples. Parasites were sorted into lysis buffer in 96-well plates using an Influx cell sorter (BD Biosciences) with a 70 µm nozzle. Sorted plates were spun at 1,000 g for 10 s and placed immediately on dry ice. 

### Library Preparation and Sequencing

Reverse transcription, PCR, and library preparation were performed as detailed previously (Reid et al., 2018; Howick et al., 2019) using a modified Smart-seq2 (Picelli et al., 2014) protocol with a non-anchored oligoDT and 25 PCR cycles. Libraries were multiplexed up to 384 and sequenced on a single lane of HiSeq 2500 v4 with 75 bp paired-end reads.

### scRNA-seq Mapping and Analysis

#### Mapping and Read Counting of Single-Cell Transcriptomes

Transcriptomes were mapped using HISAT2 (v 2.0.0-beta) (Kim et al., 2015) to the *P. berghei* v3 genome (https://www.sanger.ac.uk/resources/downloads/protozoa/, October 2016) using default parameters and summed against genes HTseq (v 0.6.0) (Anders et al., 2015) as described in (Reid et al., 2018).

#### Quality Control and Normalization

Low quality cells were identified based on the distribution of total reads and number of genes detected per cell (**Figure S1**). Cells with fewer than 500 genes per cell and 10,000 reads per cell were removed. Additionally, cells with greater than 3,000 genes per cell were removed from the *Pb single-gut data set.* Transcriptomes were normalized by calculating the base 2 logarithm of the counts per million (CPM) for each cell.

#### Parasite Stage Assignment and Pseudotime of the Pb Vector Species Data Set

Dimensionality reduction was performed using PCA in scater (v 1.16.0) based on the log CPM expression values of the top 500 variable features (McCarthy et al., 2017). To separate potential different developmental stages, we used k-means clustering using SC3 (v 1.16.0) (Kiselev et al., 2017) to assign each cell to a cluster (**Table S1**). Clusters were assigned cell-types based on marker gene expression and by using scmap (v 1.10.0) (Kiselev et al., 2018) *via* the scmapCell() function with the Malaria Cell Atlas *P. berghei* Smart-seq2 data as a reference index (Howick et al., 2019). Pseudotime was calculated using untransformed counts in monocle (Trapnell et al., 2014). For this, we selected the unsupervised procedure “dpFeature” and selected genes that are expressed in at least 10% of all cells. 528 genes with qval<0.01 were selected as ordering genes for the pseudotime.

#### Parasite Stage Assignment and Pseudotime of the Pb Single-Gut Data Set

Dimensionality reduction was performed using tSNE in scater (v 1.16.0) based on the log CPM expression values of the top 500 variable features (McCarthy et al., 2017). We then used scmap (v 1.10.0) (Kiselev et al., 2018) to assign parasite stage and order cells in developmental time. The pseudotime value and parasite stage of the matched cell in the reference Malaria Cell Atlas data was given to the query cell in the single gut ookinete data.

#### Analysis of Development-Independent Gene Expression Variability

To identify genes that varied in expression independently of developmental progression, we used a generalized linear model to regress out the effect of pseudotime as described in (Howick et al., 2019). We then used M3Drop (Andrews and Hemberg, 2018) on this corrected expression matrix to identify genes that still showed a variable pattern of expression (qval < 0.05). This method was used prior to differential expression analysis as a feature selection process to reduce the signal to noise ratio and identify genes that are likely to be biologically relevant. To identify genes that were variable independent of the midgut environment and could be potential bet-hedging genes, we used this method in the *Pb single-gut data set* by selecting features that were highly variable within each gut and identifying the intersection of the highly variable features across all four guts.

#### Differential Expression

Differential expression between ookinetes from different vector species and across individual vector guts was performed in monocle (v 2.16.0) using the differentialGeneTest function (Trapnell et al., 2014). To control for the effect of development, we included pseudotime in both the full and reduced model formulas. Only features that were expressed in more than ten cells and were identified as highly variable were included.

### Bulk RNA-seq Mapping and Analysis (Pf Bulk Data Set)

#### Mapping

Transcriptomes from individual *Anopheles* midguts infected with *P. falciparum* from a previous study (Dahalan et al., 2019) were mapped to the *P. falciparum* v3 genome (http://www.genedb.org/Homepage/Pfalciparum, October 2016) with HISAT2 (v 2.1.0) (Kim et al., 2015) using *hisat2 –rna-strandness RF –max-intronlen 5000 p 12.* Reads were summed against transcripts using featureCounts in the Subread package (v 2.0.0) (Liao et al., 2014) using *featureCounts -p -t CDS -g transcript_id -s 2*. Two samples that had fewer than 5,000 reads were removed.

#### Bulk Deconvolution

The proportion of different parasite stages in each bulk sample was estimated using CIBERSORTx (Newman et al., 2019). A signature matrix was built using *P. falciparum* ookinete, gametocyte and asexual scRNA-seq data from (Reid et al., 2018; Real et al., 2020). Cell fractions in each bulk sample were imputed based on 100 permutations.

#### Identification of Differentially Expressed and Highly Variable Genes

A minimal pre-filtering of features was performed to keep only transcripts that have at least 10 reads in total. We then performed differential gene expression analysis using Deseq2 (Love et al., 2014) with a design that included mating status, antibiotic treatment, and the proportion of ookinetes as factors in the model.

Highly variable features were identified using the Brennecke method (Brennecke et al., 2013) in M3drop (Andrews and Hemberg, 2018) after correcting the expression matrix for the proportion of ookinetes in each sample using a generalized linear model.

## Results

### Ookinetes Adjust Gene Expression Dependent on Vector Species

**Figure 1:**
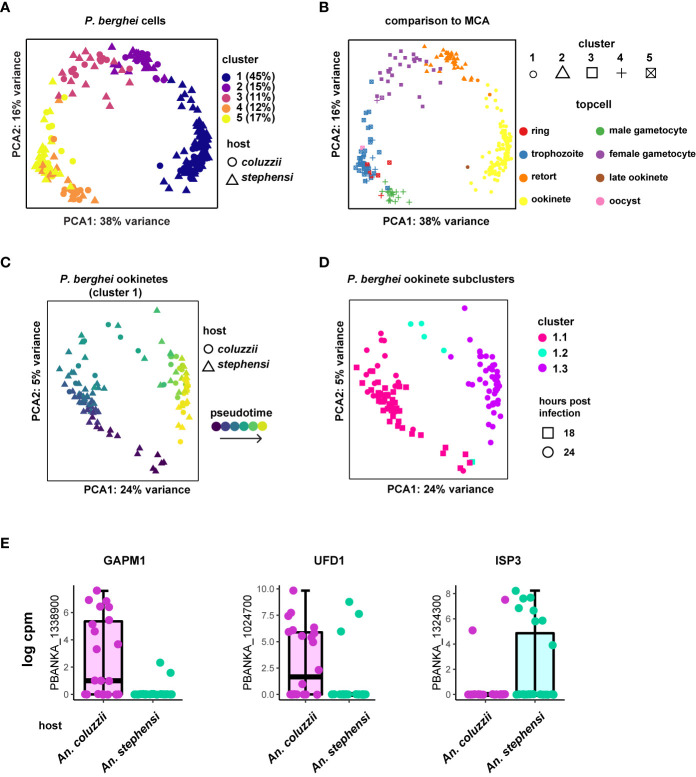
Approach to find differentially expressed genes in *P. berghei* ookinetes dependent on vector species. **(A)** Five cell clusters were identified according to marker genes for the cells collected from *An. stephensi* and *An. coluzzii* midguts, with cluster 1 representing mature ookinetes, based on marker genes expressed in this cluster (**Figure S2**, **Table S1**). Percentage of total cells for each cluster are indicated. **(B)** Cells were staged using the Malaria Cell Atlas (MCA) data as a reference index for scmap-cell. Cluster 1 represents mature ookinetes. Each stage is indicated in different colors (topcell). **(C)** Ookinetes from cluster 1 with pseudotime-corrected gene expression show additional details. **(D)** Ookinetes from cluster 1 can be clustered into three additional clusters after pseudotime correction. Cluster 1.3 was used for the analysis to investigate the effect of vector species on gene expression, as it mostly contained the ookinetes that were collected 24 h post feeding. **(E)** Selection of the three most prominent ookinete genes that are variantly expressed depending on vector host. GAPM1 (glideosome associated protein with multiple membrane spans 1), also known as PSOP23, ISP3 (inner membrane complex sub-compartment protein 3) and UFD1 (ubiquitin fusion degradation protein).

In order to understand if ookinetes can respond to the host environment, we compared single-cell transcriptomes collected from *An. coluzzii* and *An. stephensi* midgut blood boli using our *Pb vector species data set*. Cells originating from *An. stephensi* midguts are originally from the Malaria Cell Atlas data set (Howick et al., 2019) and were collected 18 and 24 h after mosquito infection. Cells collected from *An. coluzzii* midguts were collected only at 24 h after mosquito infection. Out of 286 single-cells sequenced, 253 passed quality control (**Table 1**, **Figure S1**). Parasite transcriptomes were visualized using the first two components of principal components analysis (PCA) (**Figure 1**). K-means clustering identified 5 clusters of cells based on gene expression profile (**Figure S2**). Clusters were assigned cell-types based on marker gene expression and mapping to the Malaria Cell Atlas data. Seven hundred fifty-nine marker genes were identified across all clusters and visual inspection of the top twenty marker genes for each cluster revealed that mature ookinetes belong to cluster 1, with the ookinete-specific *Pbs25*, *soap*, *cap380* and *warp* genes among the marker genes with a total of 114 cells (**Table S1**). Additionally, we staged the data using the Malaria Cell Atlas (Howick et al., 2019) as a reference index for scmap (Kiselev et al., 2018). We found that cluster 1 represents ookinetes, while the other four clusters represented a variety of different stages including asexual blood stages, male gametocytes, (unfertilized) female gametocytes, and possible retorts (**Figure S2**, **Figure 1B**).

For the remainder of our analyses, we focused only on cluster 1 cells that we identified as ookinetes (30 from *An. coluzzii* and 84 from *An. stephensi*). To control for developmental differences within the identified ookinetes, we first calculated pseudotime and then corrected the expression values using a generalized linear model with pseudotime as a covariate in the model (**Figure 1C**). Indeed, using this approach revealed that the ookinetes can be further divided into three subgroups (i.e. cluster 1.1, cluster 1.2 and cluster 1.3) (**Figure 1D**). We continued our analysis with cluster 1.3 that contained 49 mature ookinetes from both mosquito species (27 from *An. stephensi* and 22 *An. coluzzii*) as they represented the most mature ookinetes according to pseudotime and had the highest proportion of cells that were collected at 24 h. To identify genes that had an altered expression profile based on vector species, we first identified 159 genes to be highly variably expressed using M3drop (qval < 0.05) (**Table S2**). We used these 159 genes as an input to identify differentially expressed genes based on mosquito host species with pseudotime as a covariate in the model. We found 11 genes that appeared to be differentially expressed depending on the mosquito species the parasite was sampled from (qval < 0.01) (**Table S2**). Expression profiles of all 11 genes are shown in **Figures S3**, **S4**. While some differential expression of genes may be driven by expression in a small number of cells, most genes show a robust bias towards one vector species that is independent of development. One of the most prominent examples is PBANKA_1338900, the putative glideosome associated protein with multiple membrane spans 1 (GAPM1) also known as PSOP23 (Ecker et al., 2008) (**Figure 1E**). GAPM1 is predominantly expressed in ookinetes collected from *An. coluzzii* midguts but nearly completely absent in the context of an *An. stephensi* midgut. In *P. falciparum* erythrocytic stages, GAPM1 is part of the inner membrane complex (IMC) and seems to be essential for asexual proliferation in both *P. falciparum* and *P. berghei* (Bullen et al., 2009). The ubiquitin fusion degradation protein (UFD1) (PBANKA_1024700) shows a very similar pattern being predominantly expressed in ookinetes collected from *An. coluzzii* midguts, suggesting ubiquitination could support differential protein regulation between the two species. Interestingly, the inner membrane complex sub-compartment protein 3 (ISP3) (PBANKA_1324300), another IMC-related protein (Kono et al., 2012) is almost exclusively expressed in ookinetes originating from *An. stephensi* midguts.

Taken together, although the differences are mainly subtle, we find two IMC-related genes that are significantly differentially expressed in ookinetes depending on the species of the vector host, suggesting the machinery supporting ookinete motility and invasion may be able to display adaptive features in response to a varying vector species.

### Ookinetes Display Variant Gene Expression Depending on Mosquito Individual

**Figure 2:**
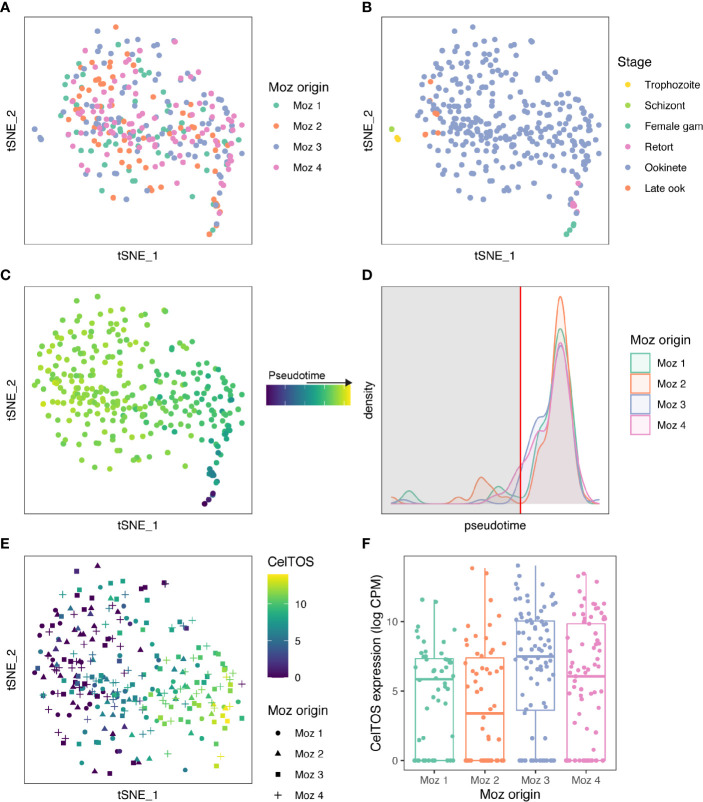
Ookinetes display variant gene expression depending on the mosquito individual. **(A)** A tSNE of 310 high-quality cells collected from four *An. stephensi* blood boli at 24 h after infectious blood meal. Cells are colored by individual mosquito origin. **(B)** Cells were staged using the Malaria Cell Atlas data as a reference index for scmap-cell. Cells that mapped to asexual stages (trophozoites and schizonts) were removed for further analyses. **(C)** 304 cells that matched ookinetes, possible retorts, or female gametocytes were ordered in pseudotime based on their scmap-cell assignment from the Malaria Cell Atlas. **(D)** A density plot of the distribution of cells over pseudotime from each mosquito. There was no difference in ookinete maturation across the 4 individual mosquitoes (ANOVA, *p* > 0.05). Immature ookinetes were removed based on the distribution for further analysis (cut off at red vertical line). In total, 275 mature ookinetes were used for further analysis. **(E)** CelTOS was one of 12 genes identified as a highly variable gene across all cells and was differentially expressed across the four midguts. Expression of CelTOS (log CPM) is shown on the tSNE. **(F)** A boxplot of CelTOS expression (log CPM) by mosquito origin. Expression of all 12 genes differentially expressed across midguts can be found in **Figure S6** and **S7**.

Above, we identify differences in ookinete gene expression based on the species of mosquito from which the ookinetes were sampled. We next addressed whether ookinetes exhibit differences in gene expression when infecting different mosquito individuals all from the same mosquito species. Here, we used the *Pb single-gut data set* comprising 310 *P. berghei* single-cell transcriptomes that were collected from four individual *An. stephensi* midgut boli 24 h after infection. Initial t-SNE analysis did not reveal any clustering of cells based on midgut origin (**Figure 2A**) suggesting that ookinetes tend to display overall largely consistent gene expression between different mosquito individuals from the same species. To further investigate more subtle patterns of expression differences, we staged the ookinetes using the Malaria Cell Atlas data (Howick et al., 2019) as a reference index for scmap (Kiselev et al., 2018). 86% of cells mapped to ookinetes (**Figure 2B**), suggesting a much more homogenous population of parasites compared to the *Pb vector species data set*. Additional comparison of the two data sets with PCA confirmed the vast majority of single-gut ookinetes overlap with cluster 1 from the *vector species data set* (**Figure S5**). Cells that mapped to asexual stages (trophozoites and schizonts) were removed from further analyses (**Figure 2B**). We next assigned a pseudotime value to each cell based on the scmap assignment and found that there were no differences in developmental timing of the parasites across the four mosquito individuals (ANOVA: *p* > 0.05, **Figures 2C, D**). We subsetted the most mature ookinetes based on the pseudotime distribution to use for further analysis (**Figures 2D, E**). Although the financial cost associated with single-cell RNA-seq limited our study to only four mosquitoes, each individual yielded more than 50 high-quality transcriptomes from mature ookinetes allowing us to understand patterns of variability both within and between vector hosts.

To identify genes that displayed different expression profiles between the midguts, we identified 682 genes that were highly variable after controlling for pseudotime. (**Table S2**). We then performed differential expression analysis on these genes and identified 12 genes that vary depending on midgut origin (qval <0.01, **Table S3**, **Figure S6**, **S7**). These 12 genes included PBANKA_1432300 (Cell-traversal protein for ookinetes and sporozoites, CelTOS) (**Figures 2E, F**) and PBANKA_1006300 (perforin-like protein 1, PLP1), which are both known to play a role in cell traversal (Ishino et al., 2005; Kariu et al., 2006). This suggests that a particular vector environment may elicit a transcriptional response, including in midgut traversal genes, which may help to maximize transmission in a context-specific manner. We also identified three genes from multigene families that were differentially expressed in ookinetes originating from individual mosquitoes: a *clag* gene (PBANKA_1400600); LAP4, an LCCL domain containing protein that regulates cell division in the oocyst (PBANKA_1319500) (Saeed et al., 2018); and a CPW-WPC containing protein (PBANKA_1245200). In asexual blood stage parasites, intra-stage variation is driven by transcriptional variation in large multigene families that are involved in host-parasite interactions (Rovira-Graells et al., 2012). For example, members of the CLAG family have been implicated in invasion, cytoadherence and nutrient uptake, which are likely to be processes where optimal function may vary across host environments (Gupta et al., 2015). Multigene family genes are often heterochromatically regulated; however, none of the differentially expressed genes we identified are from heterochromatic regions, potentially because expression of heterochromatic genes was lower in ookinetes relative to other parasite stages (**Figure S8**). Additionally, we did not find any differentially expressed ApiAP2 transcription factors (**Figure S9**), making it unclear how the identified genes are differently regulated.

### Identification of Potential Bet-Hedging Genes

**Figure 3:**
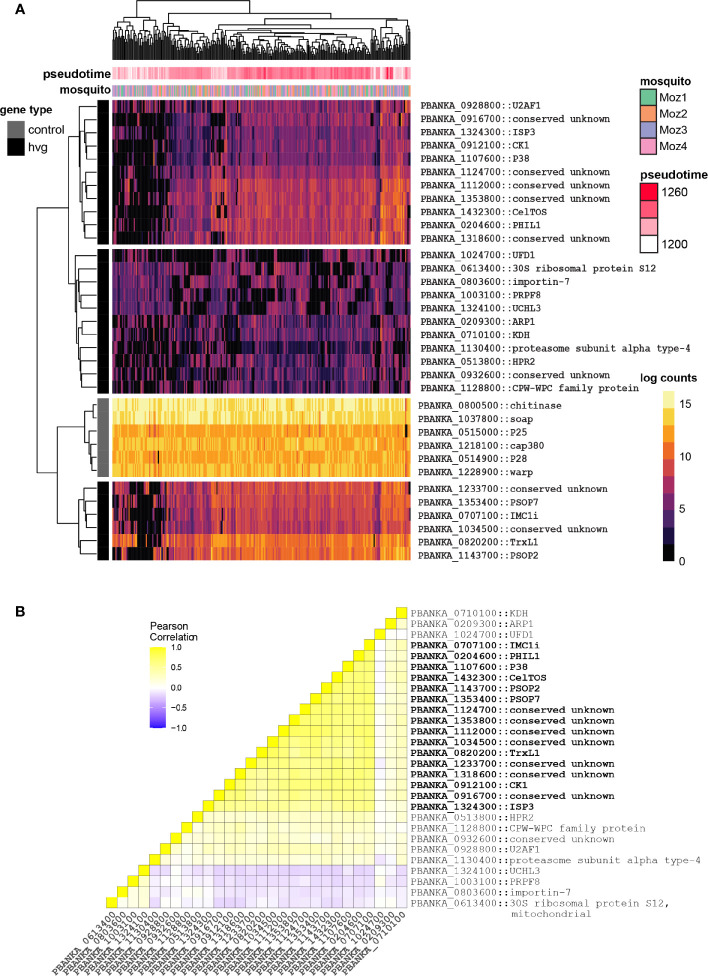
Identification of potential bet-hedging genes in ookinetes. **(A)** Heatmap of all 28 genes that are highly variably expressed in every gut from the *Pb single-gut data set*, plotted alongside six ookinete-specific genes as comparison. Each row represents a gene, and its short name is indicated if available. Values represent pseudotime-corrected log counts to control for developmental expression patterns. Black and grey lines indicate if the genes are from the highly variant gene set (hvg) or from the control gene set. For each cell, the mosquito origin is shown. Each ookinete’s coordinates along pseudotime (development) are indicated in pink. **(B)** Correlation heatmap of all 28 genes that are highly variably expressed in every gut from the *Pb single-gut data set*. IMC-related and apical genes are co-regulated in ookinetes and highlighted in bold.

We were next interested in identifying patterns of expression that are consistent with a bet-hedging strategy. Bet-hedging refers to an adaptive strategy that relies on pre-existing diversity within populations to survive potentially unknown environments. Single-cell RNA-seq allows us to understand what genes are transcriptionally variable in a population, which could indicate their role in this process. In contrast to the differential expression analysis where we were specifically interested in genes that had different expression patterns between midguts, here we were interested in genes that showed a similar pattern of variability independently of the midgut environment. To do this, we first corrected the ookinete gene counts for pseudotime, and then created a gene list of highly variable genes for the ookinetes within each individual mosquito (qval < 0.05) (**Table S3**). Looking at the intersection of these four lists, we found a total of 28 ookinete genes that are consistently highly variable (hvg) in all of our mosquitoes. Interestingly, we find several genes that encode proteins with a role in the inner membrane complex (IMC) such as photosensitized INA-labeled protein (PhIL1, PBANKA_0204600) (Saini et al., 2017) and the alveolin IMC1i (PBANKA_0707100) (Kaneko et al., 2015) and ISP3 (PBANKA_1324300), which localizes to the apical anterior apical end of the parasite where IMC organization is initiated (Poulin et al., 2013). In addition, CelTOS is highly variable in this gene set as well. Looking at pseudotime-corrected gene expression, we find that the majority of the 28 highly variant genes display a huge variety of expression between different ookinetes, compared to a control group of highly expressed ookinete-specific genes (**Figure 3A**). Looking at the correlation of these 28 genes, we find that some of these genes are indeed co-expressed, including several genes involved in the IMC and the apical end of the ookinete (**Figure 3B**). Functional work to understand the fitness of parasites that are co-expressing these genes would be necessary to validate their role in bet-hedging. For example, are the subset of cells expressing the identified IMC genes more likely to cross the midgut and develop as an oocyst compared to parasites that are not? Whilst this work is beyond the scope of this study, we have developed a framework that allowed for identification of patterns of expression that are consistent with bet-hedging. We found that a compelling proportion of variant genes encode IMC-related and apically localizing proteins suggesting that these genes may contribute to a bet-hedging strategy that involves the invasion machinery.

### Variation in *P. falciparum* Gene Expression Depending on Host Physiological Status and Midgut Origin

**Figure 4:**
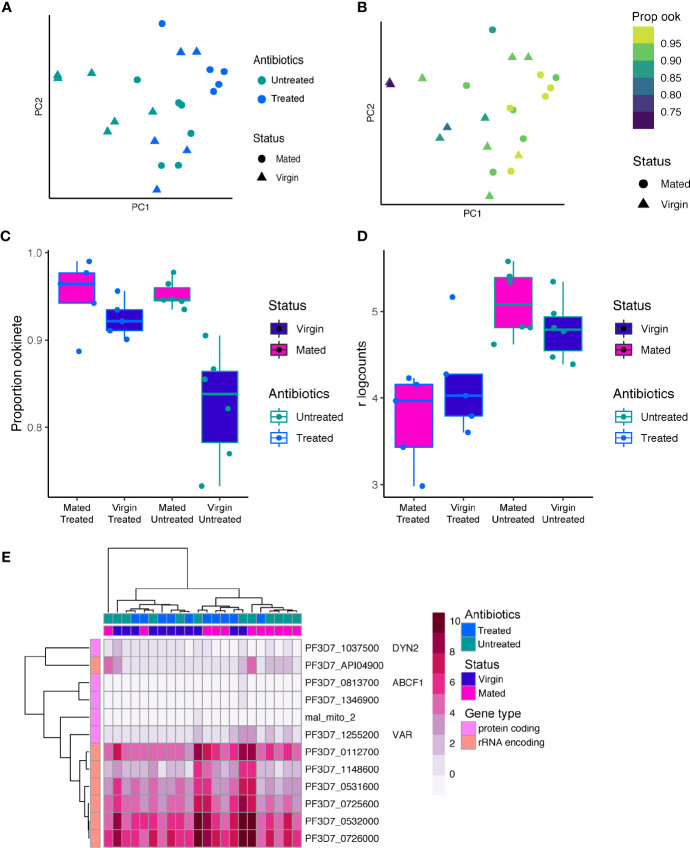
*P. falciparum* gene expression in individual midgut transcriptomes 24 h post-infection. **(A)** PCA of 22 P*. falciparum* transcriptomes from individual *Anopheles* midguts. Points are colored by antibiotic treatment and shaped by mating status. **(B)** The PCA colored by the proportion of ookinetes in each sample based on bulk deconvolution. The proportion of ookinetes increases along PC1. **(C)** A boxplot of the proportion of ookinetes in each sample by mating status and antibiotic treatment. Both antibiotic treatment, mating status, and the interaction between the two significantly impacted the proportion of ookinetes observed in each sample (ANOVA, *p* < 0.05 for each model term). **(D)** Expression of PF3D7_1216600 (CelTOS) in bulk RNAseq data from individual *An. coluzzii* midguts with blood boli. CelTOS was more highly expressed in the antibiotic untreated condition. **(E)** The 12 genes that were identified as highly variable after correcting for the proportion of ookinetes observed are shown in a heatmap of the regularized log counts from DEseq2. Many of the highly variable genes were rRNA encoding genes. Other variable genes of more moderate expression included the *var* gene PF3D7_1255200 which has been shown to be the primary *var* in transmission stages (Gómez-Díaz et al., 2017).

Although model systems, such as *P. berghei*, are key to understanding parasite biology and genetics, they may not fully capture the transcriptional variation that exists in malaria parasites infecting humans because of different biological processes and/or lack of orthology across species, especially in clonally variant gene families. To begin to understand how *P. falciparum* varies in gene expression in a host individual- and host physiological-dependent manner, we revisited the *Pf bulk RNA-seq data set* from (Dahalan et al., 2019). In this study, transcriptomes of individual *An. coluzzii* midguts 24 h after a *P. falciparum* (NF54) infectious blood meal were profiled using standard RNA-seq. The experiment was a 2x2 factorial design with both mated/virgin and antibiotic treated/untreated mosquitoes. The antibiotics included a cocktail of gentamycin, penicillin and streptomycin, which decreased the vector microbiota. The original use of the data set focused on questions around vector gene expression. Here we explore parasite gene expression from within the vector midguts. Mapping the reads to the *P. falciparum* genome revealed significant coverage of parasite genes with a mean of 2.6 x 10^4^ mapped parasite reads and 2,543 genes per gut after quality control (**Figure S1**). Principal component analysis revealed subtle separation of transcriptomes based on mating status and antibiotic treatment along PC1 and PC2 (**Figure 4A**).

To confirm that the majority of the transcriptional signature we detect at 24 h post bloodmeal originated from ookinetes, we deconvoluted the samples into cell-types using *P. falciparum* single-cell data from ookinetes, gametocytes and asexual blood stages (Reid et al., 2018; Real et al., 2020). Indeed, ookinetes made up the largest proportion of cells in all bulk samples ranging from 73 to 99% of the total composition with a median of 93% (**Figure S10**). Female gametocytes made up the second largest proportion of cells with a median of 5%. We found the proportion of ookinetes increased along PC1 (**Figure 4B**), suggesting the relative ratios of stages across the samples was driving the variation in gene expression observed. The proportion of ookinetes in each sample was also dependent on mating status and to a lesser extent antibiotic treatment and the interaction between status and antibiotic treatment (**Figures 4B, C**, **Figure S10**) (ANOVA; antibiotic treatment: *F*=8.01, *p*=0.01; mating status: *F*=21.91, *p* < 0.001; interaction: *F*=8.06 *p*=0.01). Midguts from virgin mosquitoes that were not treated with antibiotics had the lowest proportion of ookinetes, which corresponds with a lower intensity of infection in virgin relative to mated individuals seen at the oocyst stage (Dahalan et al., 2019). This suggests that the lower susceptibility seen in virgin individuals may be a result of inhibition of ookinete development instead of, or in combination with, an increased failure rate in ookinetes crossing the midgut epithelium.

We next asked if parasites showed differential expression based on their host’s mating status or antibiotic treatment. We included the proportion of ookinetes as a covariate in the design to correct for differences in expression that were a result of different cell-type ratios. We identified 2 genes differentially expressed based on mating status and 24 differentially expressed based on antibiotic treatment (FDR < 0.05) (**Figures S11**, **S12**). The set of genes that changed with antibiotic treatment were enriched for those associated with the microneme and parasite cell surface (GO:0020009, GO:0009986) Benjamini p<0.05). These included PF3D7_1216600 (CelTOS), and PF3D7_1449000 (GEST, gamete egress and sporozoite traversal protein) and PF3D7_1030900 (P28), which were all upregulated in untreated individuals (**Figure S12**). CelTOS and GEST both play a role in cell traversal at multiple stages in the life cycle (Kariu et al., 2006; Talman et al., 2011; Jimah et al., 2016), suggesting the microbiota composition may influence cell traversal strategy. CelTOS was also observed to be differentially expressed at the single-cell level in *P. berghei* between individuals, which potentially could be driven by variation in the microbiota, indicating a conserved response to the midgut environment across *Plasmodium* species.

Finally, we asked which *P. falciparum* genes show a highly variable pattern of expression across individual midguts. Using the Brennecke method (Brennecke et al., 2013), we identified 12 genes that have significant biological variation (FDR < 0.05) (**Figure 4C**, **Table S4**). Seven of these genes were highly expressed rRNA encoding genes (**Figure 4D**). The active rRNA subunits switch between the mammalian and vector host across *Plasmodium* species (Waters et al., 1989), and their expression pattern may represent inter-vector individual variation in this process driven by selection of different parasite clones or a response to the environment. We also identified a single gene PF3D7_1255200 from the clonally variant *var* gene family which encodes the virulence factor Erythrocyte Membrane Protein 1 (PfEMP1). This particular *var* gene has previously been identified as the primary *var* expressed in transmission stages originating from field samples (Gómez-Díaz et al., 2017).

## Discussion

Here, we used a combined approach of single-cell sequencing together with bulk sequencing of both *P. berghei* and *P. falciparum* ookinete stages to address variance of expression in this stage. We find that single-cell sequencing is a powerful tool to address transcriptional variance in ookinetes, a stage where the common consensus is that little to no transcriptional variance occurs. Previous studies indicated that the ookinete transcriptome is somewhat “hard-wired” and made up of a mixture of derepressed maternally inherited transcripts together with new transcripts that are controlled by the ookinete-specific transcription factor AP2-O (Yuda et al., 2009; Kaneko et al., 2015). Although the vast majority of transcriptional variation observed resulted from developmental differences in individual parasites, we developed a framework that allowed us to identify a small number of genes that do display variable expression patterns that are responsive to the host condition.

We identify differentially expressed ookinete genes in *P. berghei* using a nested approach by identifying highly variable genes and using them as the input for further analysis in order to minimize potential noise in the data. Using this approach, we identify 11 genes showing expression dependent on vector species and 12 genes showing expression dependent on vector individuals. Additionally, we identify 28 potential bet-hedging genes, which show a variable pattern of expression independent of the vector individual. For the *P. falciparum* samples, we performed the differential expression and highly variable gene search independently due to the bulk data collection and found 2 genes showing expression dependent on vector mating status, 24 genes showing expression dependent on vector antibiotic treatment, and 12 that show a highly variable pattern of expression. Only three genes were identified in more than one analysis. UFD1 and ISP3 were differentially expressed in the *Pb vector species data set* and identified as potential bet-hedging genes in the *single-gut ookinete data set*. CelTOS was identified as both differentially expressed and a potential bet-hedging gene in the *single-gut ookinete data set.* Additionally, it was differentially expressed in *P. falciparum* in response to antibiotic treatment in the *Pf bulk data set*.

Our analysis highlighted several genes that appear to be clonally variantly expressed that are involved in invasion and located in euchromatic regions of the genome. This is in contrast to patterns of heterochromatin observed in asexual blood stages and transmission stages, where multigene families and some invasion pathways are clonally variant and regulated by heterochromatin (Flueck et al., 2009; Lopez-Rubio et al., 2009; Salcedo-Amaya et al., 2009; Crowley et al., 2011; Brancucci et al., 2014; Fraschka et al., 2018; Witmer et al., 2020). None of the genes we identified overlapped with those found to be heterochromatic regions in either *P. berghei* asexual blood stages or ookinetes (Fraschka et al., 2018; Witmer et al., 2020) (**Figure S8**). In general, we find that heterochromatic genes are largely silenced in ookinetes. In contrast, we find that in ookinetes variant gene expression is not necessarily a yes or no decision (as seen with heterochromatic genes in other life stages) but manifests as variable expression levels of non-heterochromatic genes. This is in stark contrast to how variegated gene expression is driven by heterochromatin in asexual blood stages. One limitation of this study is that clonally variant expression of preceding or subsequent stages for each experiment is unknown as we only sampled ookinetes. However, we do know that heterochromatic *pir* genes are highly variable in male gametocytes (Reid et al., 2018) and both male and females show higher expression of heterochromatic genes (**Figure S8**), but notably these data are from a different experimental infection and should be carefully interpreted. Still, the majority of the genes detected in our study are ookinete-specific and euchromatic, supporting a model of variegated expression in the invasive ookinete stage that is not driven/controlled by heterochromatin formation.

However, it is worth noting that the *var* gene we identified to be expressed in *P. falciparum* midgut samples is the same gene that was detected in *P. falciparum* oocysts from field samples (Gómez-Díaz et al., 2017) but different from the *var* gene detected in mosquito stages in another study (Zanghì et al., 2018). Although to date it is unclear why *var* gene transcripts are found in mosquito stage parasites, as the PfEMP1 proteins they encode for have a very prominent role in antigenic variation in asexual blood stages (Scherf et al., 2008). Nonetheless, this finding suggests that there may be a favoritism of expression in *var* genes in mosquito stages and that not all *var* genes are predisposed to be expressed at this stage.

In our analyses we identify some components of the IMC supporting invasion and invasion proteins themselves to be variably expressed. Remarkably, we found variation in response to the species of mosquitoes in which ookinetes develop. In the wild, parasites can encounter many vector species, for instance *P. falciparum* can infect more than 70 *Anopheles* species (Sinka et al., 2012). Our observation raises the possibility that there exists some invasion plasticity to cater for the host environment, such a phenomenon would be an important consideration in the development of transmission blocking vaccines. For example, *P. berghei* infections in *An. stephensi* are more intense (*i.e.* have more oocysts) than in *An. coluzzii* (Alavi et al., 2003). The differentially expressed genes identified here may be actively involved in modulating infection intensity or may be differentially expressed *via* selection on the parasite population. Secondly, we found invasion related variation in response to the mosquito environment within the same species suggesting that there is also fine tuning in response to more minor variations in the mosquito environment to potentially enhance transmission. A particularly interesting transcript identified in several of our analyses is CelTOS, a protein essential for invasion that actively takes part in membrane disruption during pore formation of the midgut epithelial cells in order for the ookinete to reach the basal lamina (Kariu et al., 2006; Jimah et al., 2016). Although CelTOS is essential for ookinete invasion, lysis of the enterocyte can also have a deleterious effect on epithelial integrity of the gut and vector survival. It may therefore be advantageous for the parasite to modulate such lytic factors to maximize invasion whilst limiting host death in response to environment or as a population wide bet-hedging strategy to limit the number of invasion events within a single gut. Although such hypotheses would require much deeper validation, our data is suggestive of adaptive phenotypic plasticity of the parasite invasion machinery in response to environmental changes.

It is important to note that this study provides a first overview of variant gene expression in individual ookinetes but is limited in the fact that it contains descriptive data. Further experimental validation is needed to confirm the individual involvement of candidate genes. Nonetheless, this study gives good insight into ookinete biology and will prove valuable for the community.

## Data Availability Statement

The data sets generated for this study can be found in ENA at: https://www.ebi.ac.uk/ena/browser/text-search?query=ERP123837.

## Ethics Statement

All animal experimental procedures were reviewed and approved by the Animal Welfare and Ethical Review Body (AWERB) and the United Kingdom Home Office. These procedures were in accordance with the Animal Scientifics Procedures Act 1986, under the UK Home Office License P64F4ADF7.

## Author Contributions

ML and VH conceived of and designed the study. VH and AT performed *P. berghei* experiments. TM performed mouse work for *P. berghei* experiments. FD performed *P. falciparum* experiments. KW and VH analyzed the data. KW, AT, VH, and ML wrote the manuscript with contributions from other authors. All authors contributed to the article and approved the submitted version.

## Funding

The Wellcome Sanger Institute is funded by the Wellcome Trust (grant 206194/Z/17/Z), which supports ML. ML is supported by an MRC Career Development Award (G1100339). VH is supported by a Sir Henry Dale Fellowship jointly funded by the Wellcome Trust and the Royal Society (grant 220185/Z/20/Z).

## Conflict of Interest

The authors declare that the research was conducted in the absence of any commercial or financial relationships that could be construed as a potential conflict of interest.
