# Supplementary material for: Using scRNA-seq to Identify Transcriptional Variation in the Malaria Parasite Ookinete Stage
Source: Front Cell Infect Microbiol. 2021 Mar 1;11:604129. doi: 10.3389/fcimb.2021.604129 (PMC7958875; doi:10.3389/fcimb.2021.604129)
Supplement: Supplementary file 1 [file DataSheet_1.docx]

Supplementary Material

# Supplementary Tables and Figures

## Supplementary Tables (Tables S1-S4)

**Table S1.** List of genes expressed in each *P. berghei* cluster from *Pb vector species data set*

**Table S2.** Highly variable and DE ookinete genes from *Pb vector species data set*

**Table S3.** Highly variable and DE ookinete genes from *Pb single-gut data set*

**Table S4**. Differentially expression and highly variable genes in *Pf bulk data set*

## Supplementary Figures (Figures S1-S12)


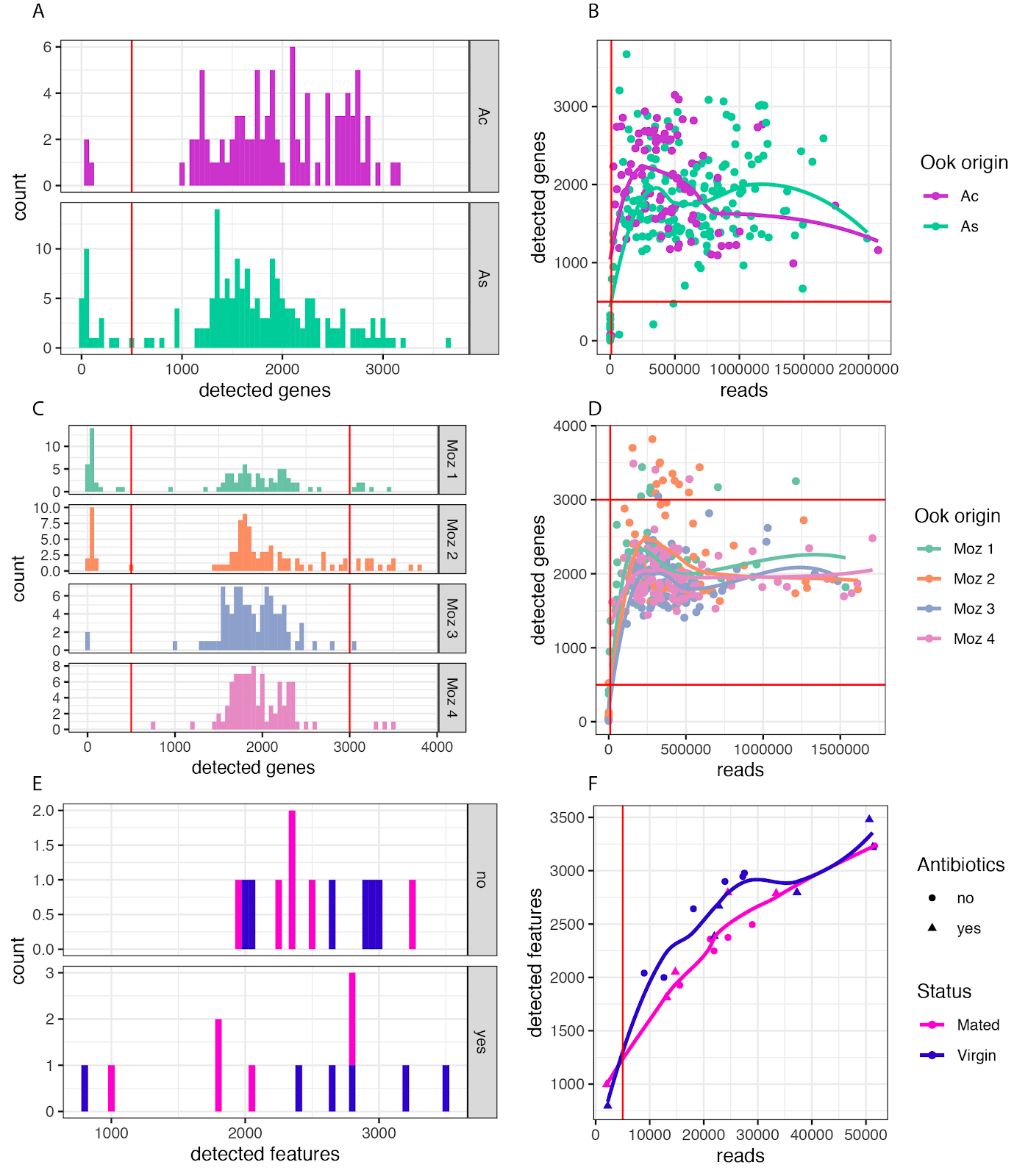


**Figure S1. Quality control of RNA-seq data.** Single-cell datasets were filtered based on the number of reads and genes detected per cell (A-D). Bulk RNA-seq data was filtered based on the number of reads per sample. **A.** The distribution of genes per cell faceted by vector species origin (Ac: *An. coluzzii*, As: *An. stephensi*) from the *Pb vector species dataset*. **B.** The number of reads per cell plotted against the number of detected genes in the *Pb vector species dataset*. Cells with fewer than 10,000 reads or 500 genes detected were removed from further analysis. **C.** The distribution of genes per cell faceted by individual *An. stephensi* mosquito origin from the *Pb single-gut dataset*. **D.** The number of reads per cell plotted against the number of detected genes in the *Pb single-gut dataset*. Cells with fewer than 10,000 reads or 500 genes detected, or with more than 3000 genes detected were removed from further analysis. **E.** The distribution of *P. falciparum* features (transcripts) per cell faceted by antibiotic treatment from the *Pf bulk dataset*. **F.** The number of *P. falciparum* reads per sample plotted against detected features in the *Pf bulk dataset*. Samples with fewer than 5,000 reads were removed from further analysis.


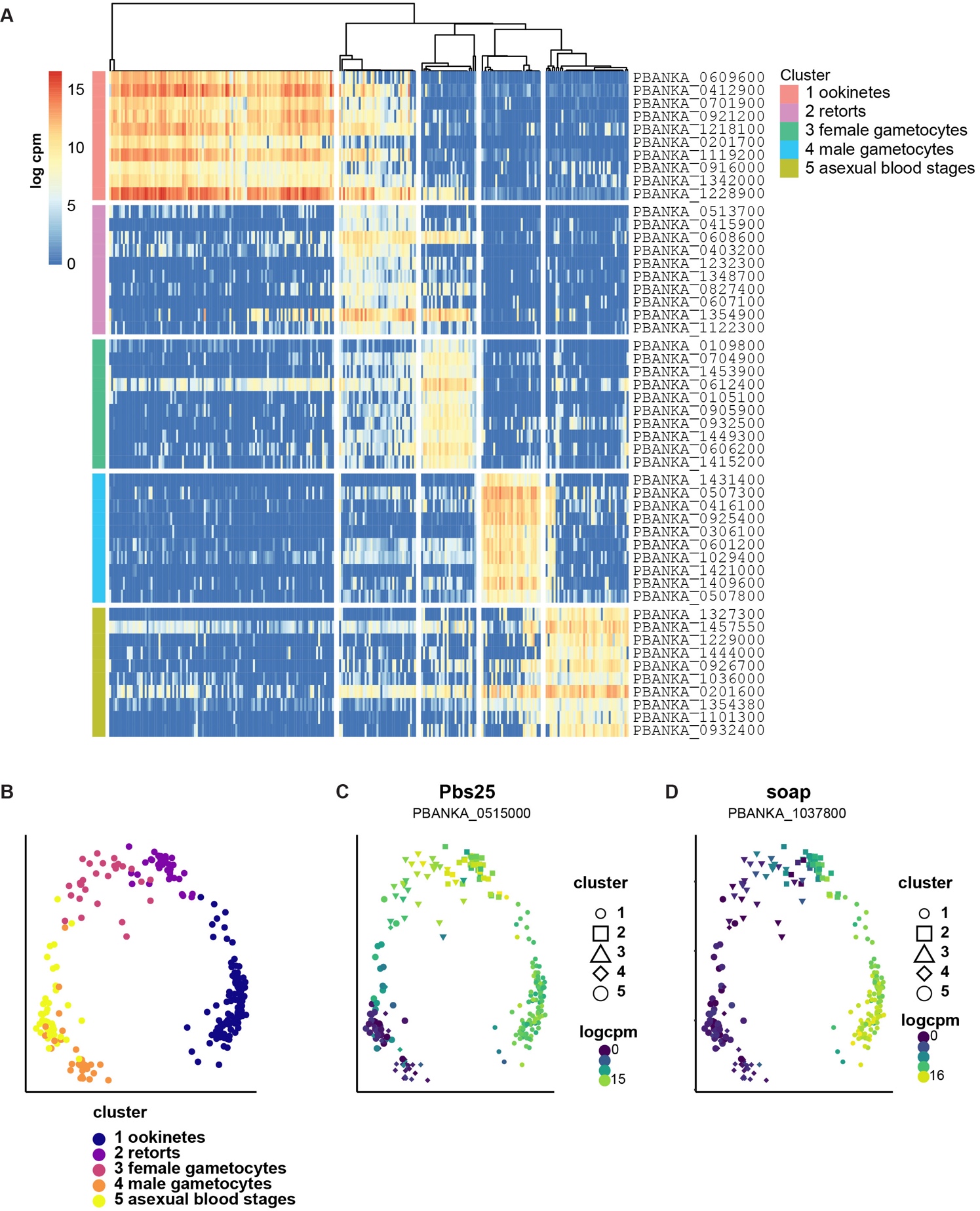


**Figure S2. Cells retrieved from *An. stephensi* and *An. coluzzii* midguts form 5 clusters using K-means. A.** The genes with the area under the ROC curve (AUROC) > 0.85 and with the p-value < 0.01 are defined as marker genes and the top 10 marker genes of each cluster are visualized in this panel. All markers can be found in **Table S1**. Cluster 1 represents mature ookinetes, with marker genes such as cap380 [(Srinivasan et al., 2008)](https://paperpile.com/c/rOiTXe/Tt0fk) and WARP [(Yuda et al., 2001)](https://paperpile.com/c/rOiTXe/wCJVP) among others. Marker genes for cluster 2 include AP2-O5 [(Zhang et al., 2017)](https://paperpile.com/c/rOiTXe/PF6c6) and CSP [(Dame et al., 1984)](https://paperpile.com/c/rOiTXe/tjahl),  both transmission-associated genes, however these cells were missing some ookinete-marker genes, indicating that they were possible retorts and were excluded from further analysis. Cluster 3 most probably represents unfertilised females as one of the marker genes is AP2-O [(Yuda et al., 2009)](https://paperpile.com/c/rOiTXe/koE8e), the transcript of which is translationally repressed in female gametocytes [(Mair et al., 2006)](https://paperpile.com/c/rOiTXe/0CJLy). Cluster 4 most likely represents male gametocytes, as one of the marker genes is PBANKA_0416100, which is known to be male gametocyte-specific [(Howick et al., 2019)](https://paperpile.com/c/rOiTXe/RIJUH). Marker genes for cluster 5 include plasmepsin IV [(Spaccapelo et al., 2010)](https://paperpile.com/c/rOiTXe/T6mwU) and an ETRAMP [(Spielmann et al., 2003)](https://paperpile.com/c/rOiTXe/ohxkG), both of which are expressed in asexual blood stage parasites. Based on these findings, we retained cluster 1 (114 cells) as ookinetes, as it contained cells that highly express ookinete-specific genes and continued with further analysis. **B.** Same as Fig 1A, showing the 5 cell clusters and the cells they represent. **C.** expression of the ookinete-specific gene *Pbs25* in each cluster. **D.** Expression of the ookinete-specific gene *soap* in each cluster.


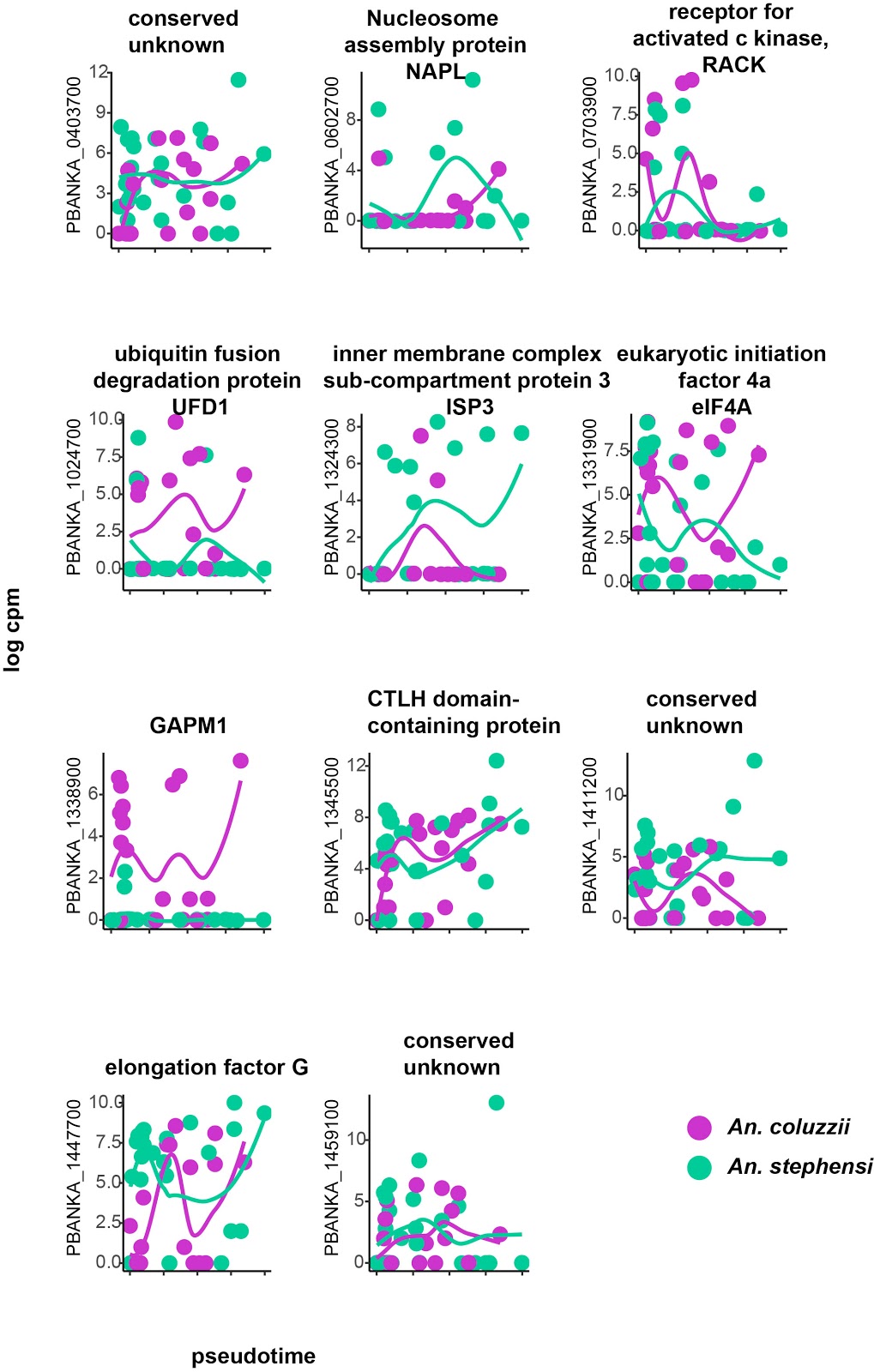


**Figure S3. 11 genes are differentially expressed in *P. berghei* ookinetes depending on host species.** Jitter plots showing expression for each gene dependent on pseudotime. Expression is shown in log cpm. Purple denotes ookinetes collected from *An. gambiae*, and green denotes ookinetes collected from *An. stephensi*.


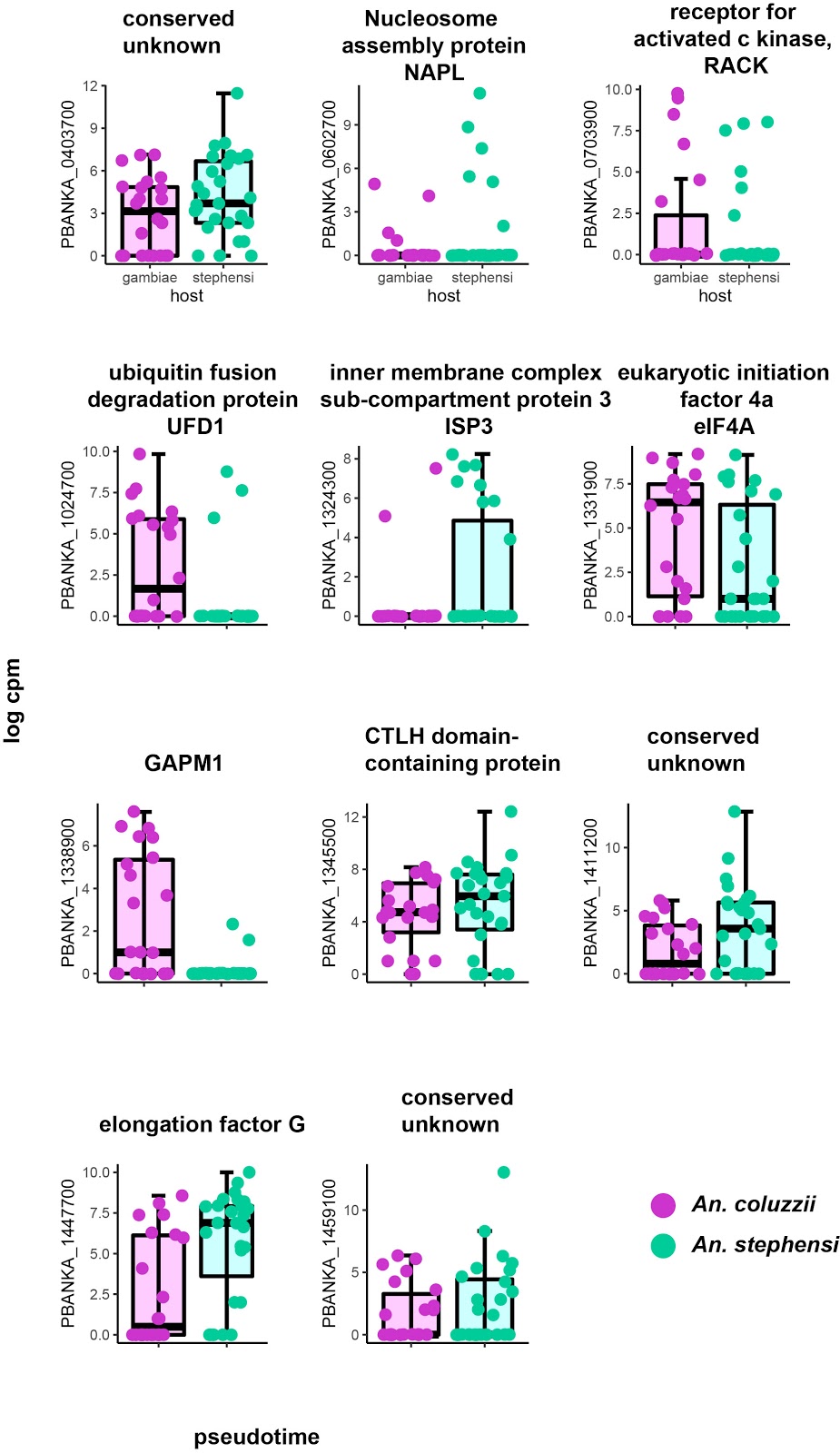


**Fig S4. 11 genes are differentially expressed in *P. berghei* ookinetes depending on host species.**

Boxplots of the genes found to be differentially expressed in *P. berghei* ookinetes depending on host species. Expression is shown in log cpm. Purple denotes ookinetes collected from *An. coluzzii*, and green denotes ookinetes collected from *An. stephensi*.


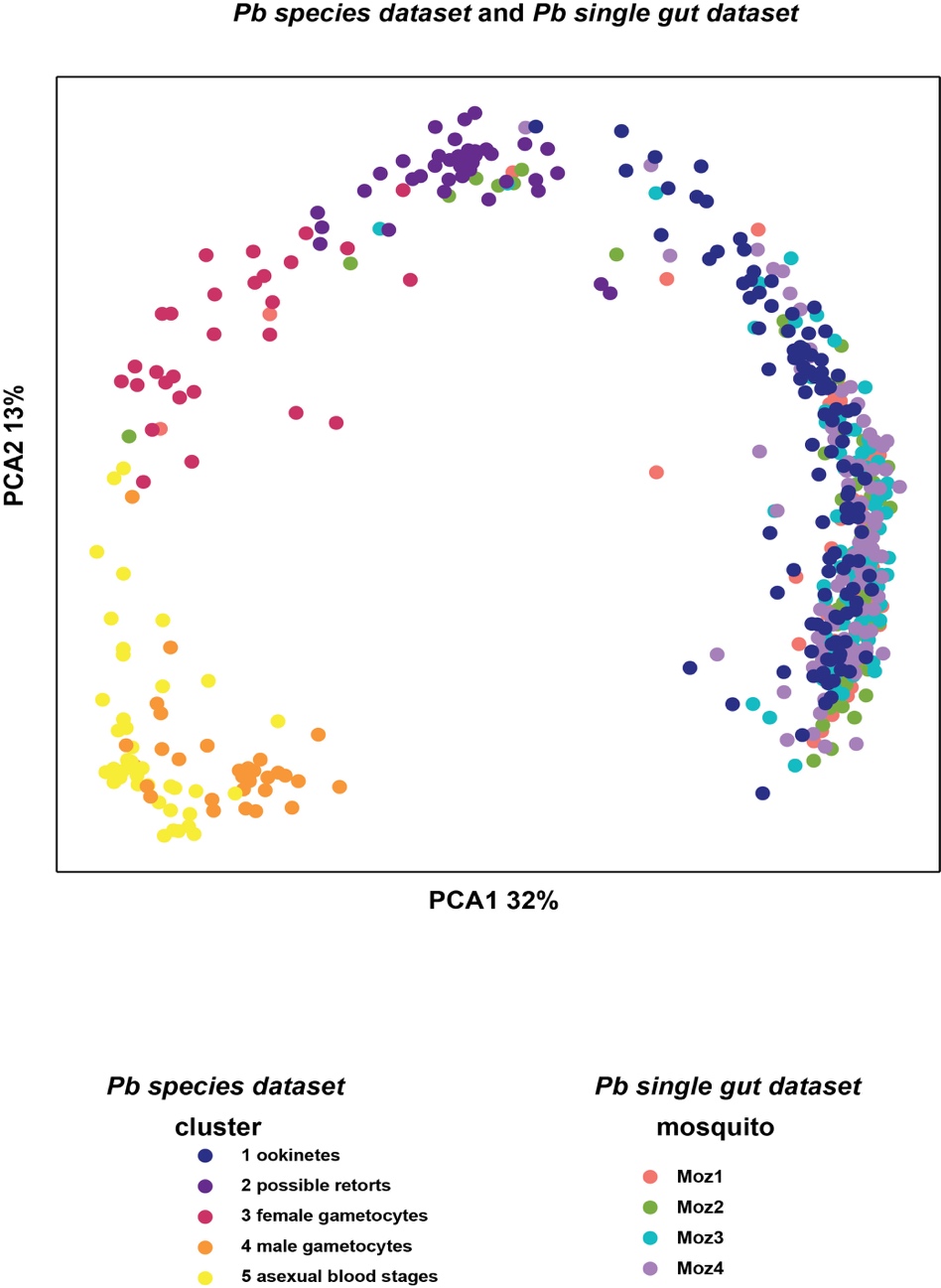


**Figure S5. PCA comparing the *Pb species dataset* to the *Pb single-gut dataset.*** Cells are coloured according to the cell cluster from the *Pb vector species dataset*, or the mosquito origin from the *Pb single-gut dataset*.


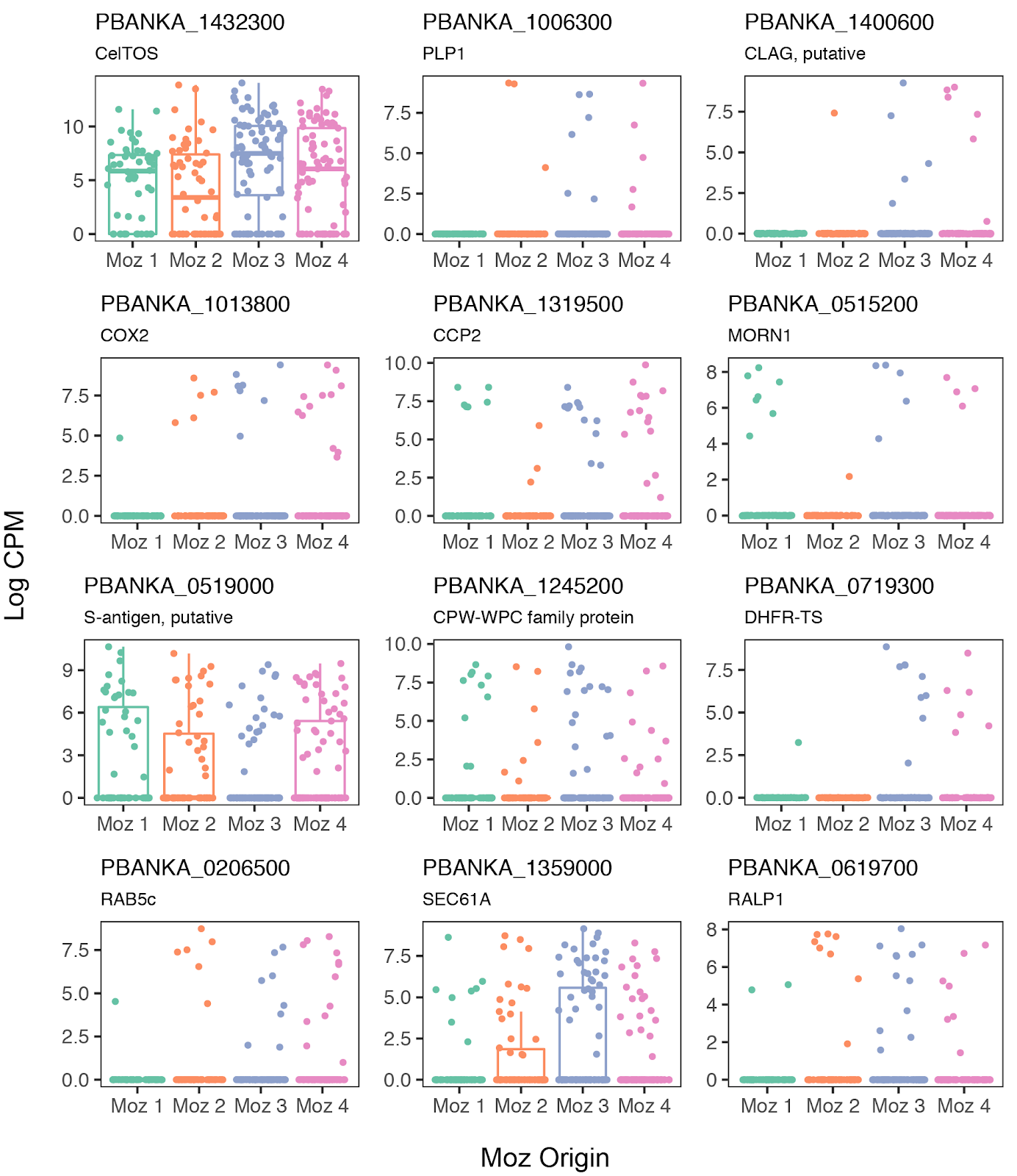


**Figure S6. Differential expressed genes in single gut ookinetes.** Boxplots of the 12 *P. berghei* genes that were identified as differentially expressed across the 4 individual midguts.


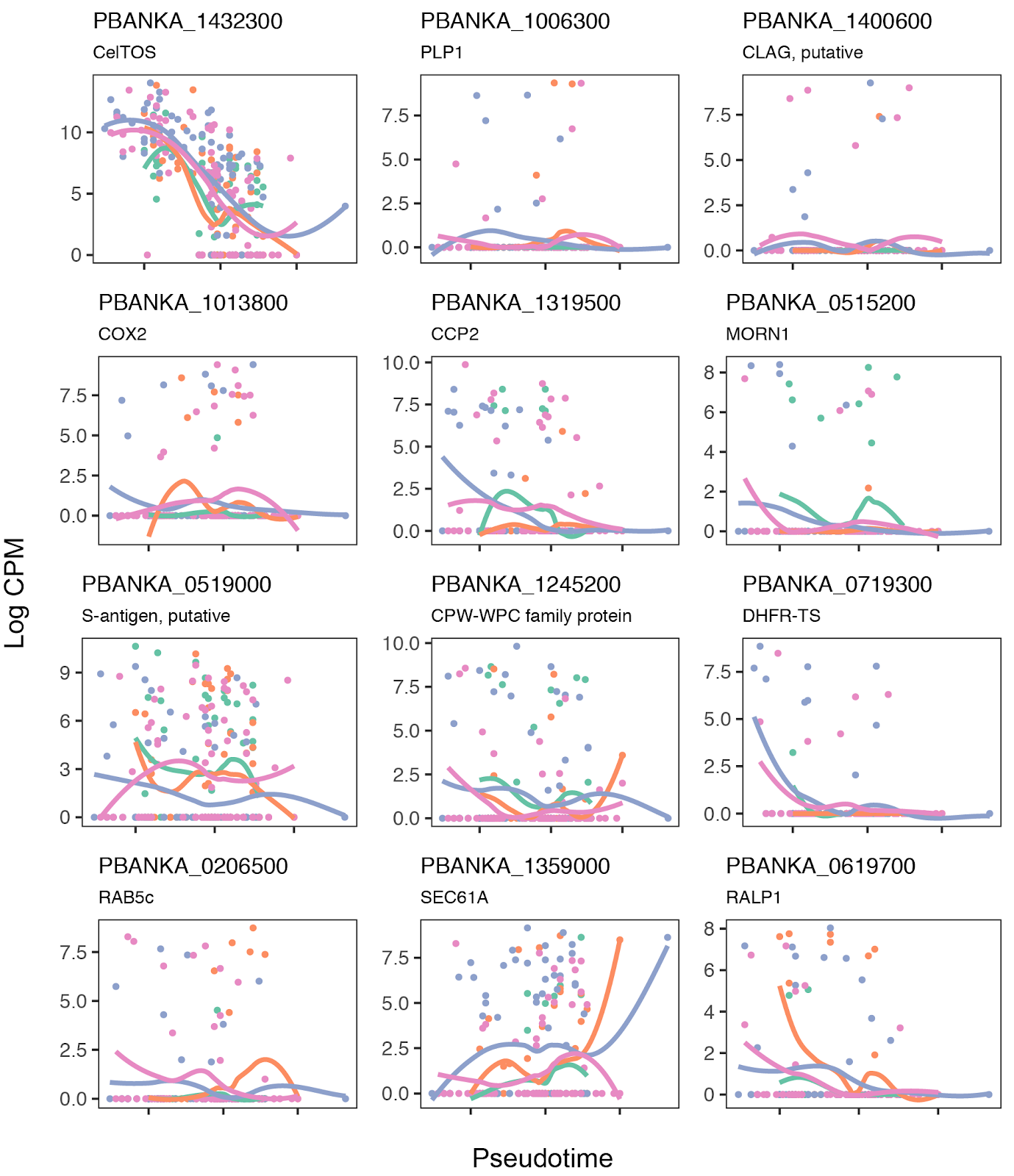


**Figure S7. Differentially expressed genes in single gut ookinetes plotted over pseudotime.** Expression of the 12 genes differentially expressed across individual midguts over pseudotime coloured by midgut of origin. Differences in development were controlled for by selecting cells that mapped to mature ookinetes and additionally including pseudotime as a covariate in the differential expression analysis.


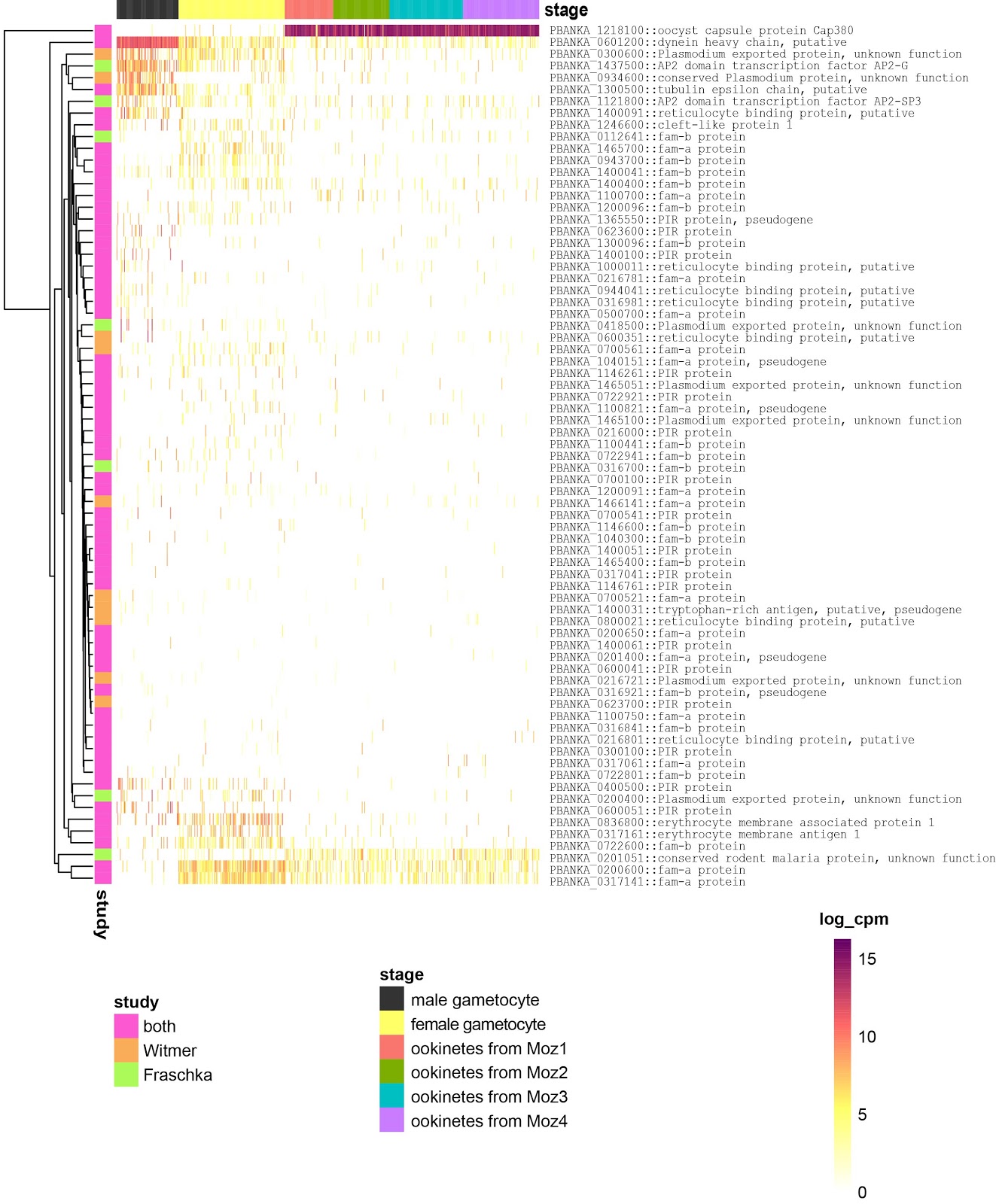


**Figure S8. Expression of heterochromatic genes in single-gut ookinetes.** Of the 233 genes deemed heterochromatic by HP1-occupancy from two studies (Fraschka et al., 2018, Witmer et al., 2020), 73 genes were detected in the single gut dataset.  Male and female gametocytes from [(Howick et al., 2019)](https://paperpile.com/c/rOiTXe/RIJUH) are included for comparison. In general, we observed lower expression of heterochromatic genes in ookinetes relative to gametocytes.

**
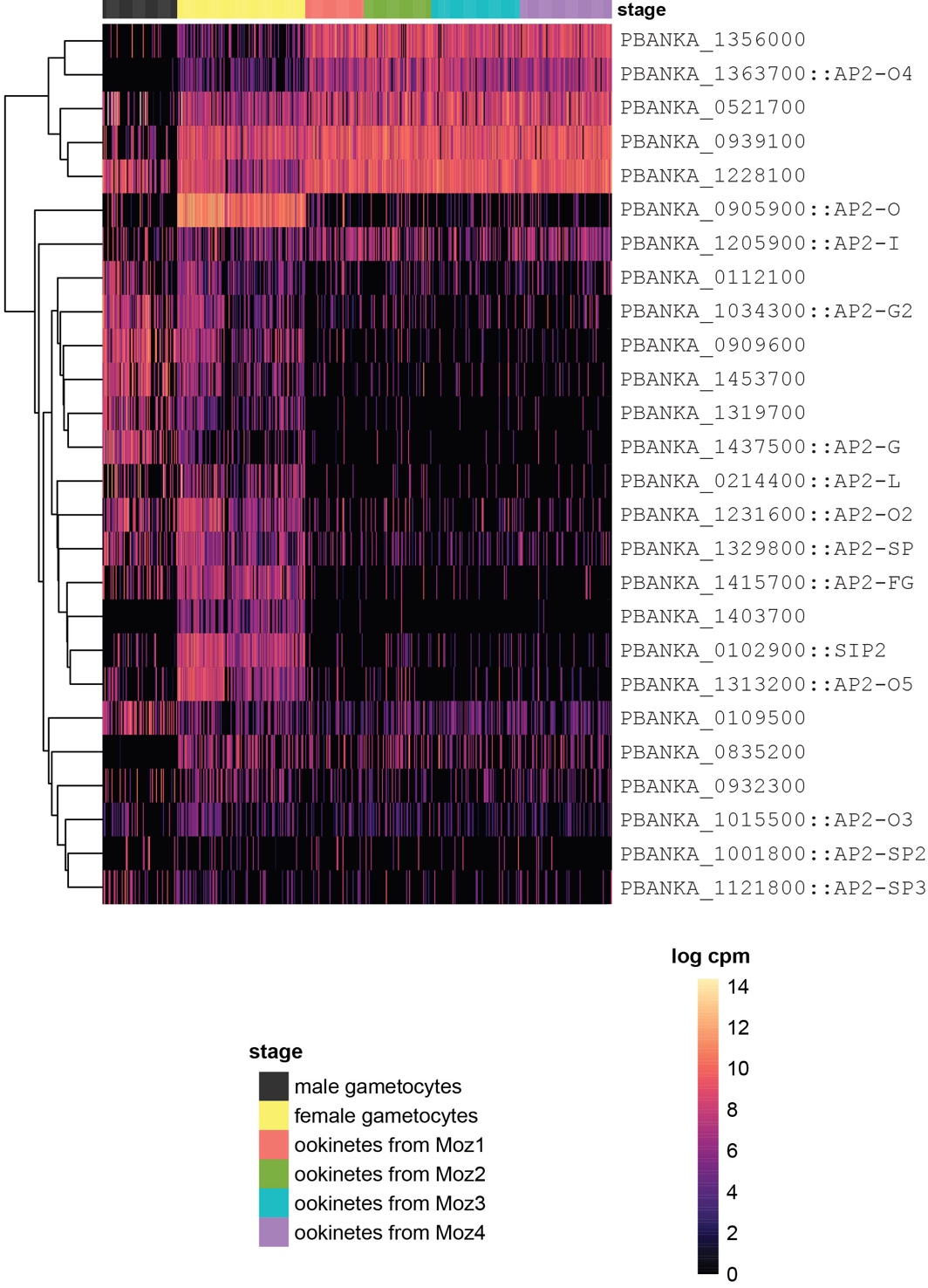
**

**Figure S9. Expression of ApiAP2 transcription factors in single-gut ookinetes.**

Expression of all 26 *P. berghei* ApiAP2 genes in the single-gut dataset. Male and female gametocytes from [(Howick et al., 2019)](https://paperpile.com/c/rOiTXe/RIJUH) are included for comparison.


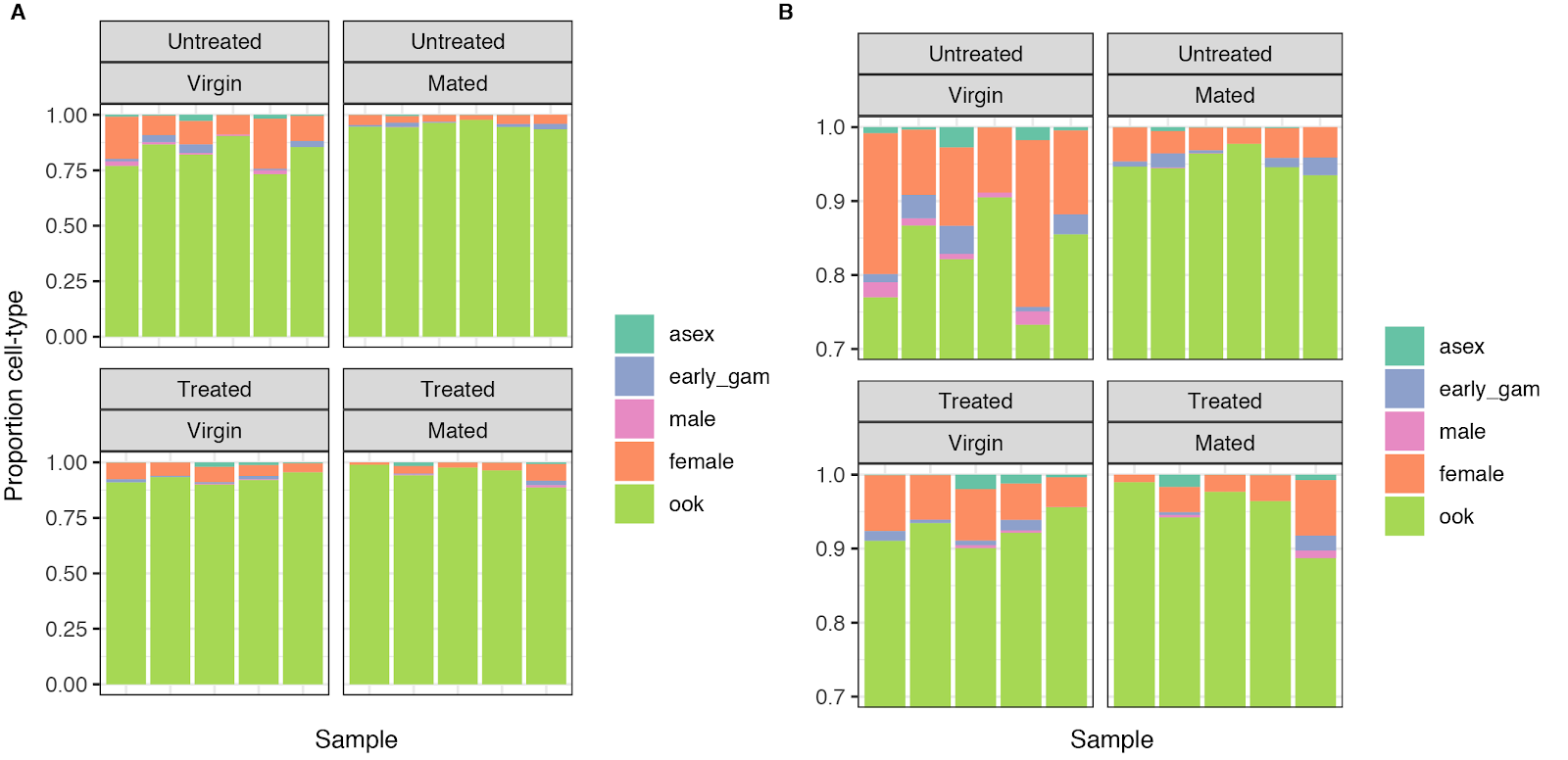


**Figure S10. Deconvolution of bulk RNA-seq using single-cell data.** Using single-cell RNA-seq data from *P. falciparum* transmission stages [(Real et al., 2020)](https://paperpile.com/c/rOiTXe/V3rf8), we deconvoluted each bulk RNA-seq sample into cell-type proportions. **A.** We found that the majority of signal from each sample was coming from ookinetes. **B.** We observed differences in the proportion of ookinetes depending on mating status and antibiotic treatment with antibiotic-untreated virgin midguts having the lowest proportion of ookinetes.


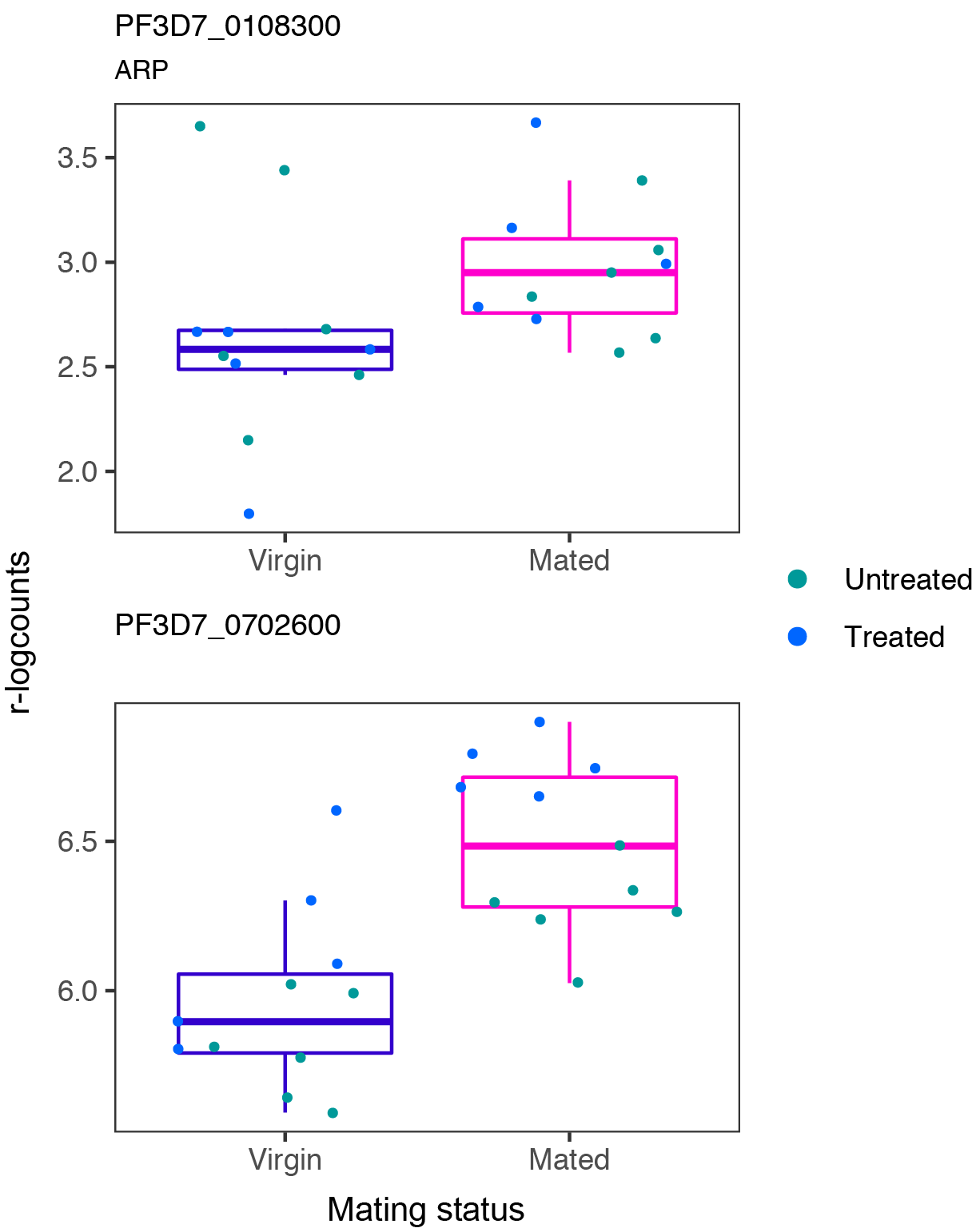


**Figure S11.  Differential expression of ookinete genes depending on mating status of the host.**Expression of two *P. falciparum* genes (regularized log counts) that were identified as differentially expressed (FDR < 0.05) in mated vs virgin from individual midgut plus blood bolus bulk RNA-seq.


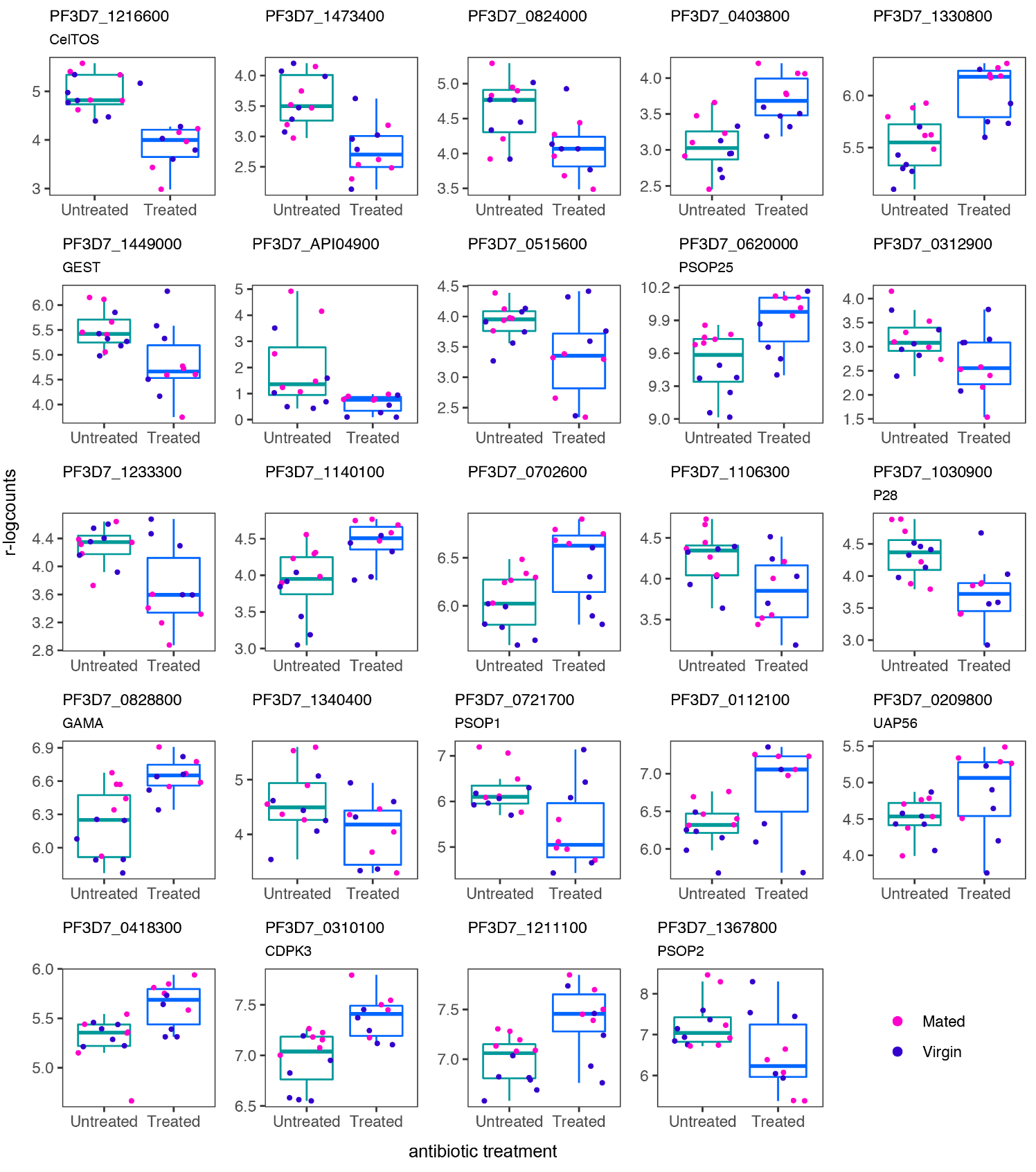


**Figure S12.  Differential expression of ookinete genes depending on antibiotic treatment of the host.**Expression of 24 *P. falciparum* genes (regularized log counts) that were identified as differentially expressed (FDR < 0.05) in antibiotic treated vs untreated from individual midgut plus blood bolus bulk RNA-seq.

**Supplementary References**

[Dame, J. B., Williams, J. L., McCutchan, T. F., Weber, J. L., Wirtz, R. A., Hockmeyer, W. T., et al. (1984). Structure of the gene encoding the immunodominant surface antigen on the sporozoite of the human malaria parasite Plasmodium falciparum. *Science* 225, 593–599.](http://paperpile.com/b/uv1y5U/nDISQ)

[Mair, G. R., Braks, J. A. M., Garver, L. S., Wiegant, J. C. A. G., Hall, N., Dirks, R. W., et al. (2006). Regulation of sexual development of Plasmodium by translational repression. *Science* 313, 667–669.](http://paperpile.com/b/uv1y5U/F5UBQ)

[Spaccapelo, R., Janse, C. J., Caterbi, S., Franke-Fayard, B., Bonilla, J. A., Syphard, L. M., et al. (2010). Plasmepsin 4-deficient Plasmodium berghei are virulence attenuated and induce protective immunity against experimental malaria.](http://paperpile.com/b/uv1y5U/U7sTx) *[Am. J. Pathol.](http://paperpile.com/b/uv1y5U/U7sTx)* [176, 205–217.](http://paperpile.com/b/uv1y5U/U7sTx)

[Spielmann, T., Fergusen, D. J. P., and Beck, H.-P. (2003). etramps, a new Plasmodium falciparum gene family coding for developmentally regulated and highly charged membrane proteins located at the parasite-host cell interface. *Mol. Biol. Cell* 14, 1529–1544.](http://paperpile.com/b/uv1y5U/ZmhBu)

[Srinivasan, P., Fujioka, H., and Jacobs-Lorena, M. (2008). PbCap380, a novel oocyst capsule protein, is essential for malaria parasite survival in the mosquito. *Cell. Microbiol.* 10, 1304–1312.](http://paperpile.com/b/uv1y5U/6hLum)

[Yuda, M., Yano, K., Tsuboi, T., Torii, M., and Chinzei, Y. (2001). von Willebrand Factor A domain-related protein, a novel microneme protein of the malaria ookinete highly conserved throughout Plasmodium parasites. *Mol. Biochem. Parasitol.* 116, 65–72.](http://paperpile.com/b/uv1y5U/uJBlB)

[Zhang, C., Li, Z., Cui, H., Jiang, Y., Yang, Z., Wang, X., et al. (2017). Systematic CRISPR-Cas9-Mediated Modifications of Plasmodium yoelii ApiAP2 Genes Reveal Functional Insights into Parasite Development. *MBio* 8, e01986–17.](http://paperpile.com/b/uv1y5U/Nfj74)
